# Introducing JMIR Rehabilitation and Assistive Technologies: A Venue for Publishing Interdisciplinary Research on the Development, Implementation, and Evaluation of Health Innovations and Emerging Technologies in the Field of Rehabilitation

**DOI:** 10.2196/56348

**Published:** 2024-04-22

**Authors:** Sarah EP Munce

**Affiliations:** 1 KITE Research Institute Toronto Rehabilitation Institute-University Health Network Toronto, ON Canada; 2 Occupational Science & Occupational Therapy University of Toronto Toronto, ON Canada; 3 Rehabilitation Sciences Institute University of Toronto Toronto, ON Canada; 4 Institute of Health Policy, Management & Evaluation University of Toronto Toronto, ON Canada

**Keywords:** rehabilitation, assistive technologies, *JMIR Rehabilitation and Assistive Technologies*, digital, online

## Abstract

Rehabilitation supports the affected individual and their caregivers in managing the health condition and its associated symptoms, altering the environment to accommodate needs, adapting tasks for safe and independent performance, facilitating self-management, and using assistive devices and technologies. *JMIR Rehabilitation and Assistive Technologies* focuses on pragmatic yet rigorous and impactful science that reports on the development, implementation, and evaluation of health innovations and interventions as well as emerging technologies in the field of rehabilitation.

## Background

As defined by the World Health Organization, rehabilitation is “a set of interventions designed to optimize functioning and reduce disability in individuals with health conditions in interaction with their environment” [1]. The World Health Organization elaborates that rehabilitation helps individuals of all ages become as independent as possible in daily activities and promotes meaningful participation in many aspects of life, including education, work, recreation, and looking after family. Rehabilitation enables this participation and independence by supporting the affected individual and their caregivers in addressing the health condition and its associated symptoms, altering the environment to accommodate needs, adapting tasks for safe and independent performance, supporting self-management, and using assistive devices and technologies. These strategies can help the individual and their caregiver to overcome challenges in thinking, seeing, hearing, communicating, eating, and mobilizing [1].

The benefits of rehabilitation are multifaceted and can reduce the impact of acute and chronic health conditions, illnesses, and injuries. Rehabilitation can also support other health interventions, such as medical or surgical procedures, to achieve optimal outcomes. Furthermore, rehabilitation is highly person driven, meaning that the interventions selected for each individual are tailored to their unique goals and preferences. Rehabilitation can be provided in many different settings, such as in inpatient or outpatient hospital settings, or community settings such as an individual’s home, a school, a workplace, and increasingly, remotely [1,2]. Indeed, an overview of telerehabilitation and its fields of application, with an analysis of the benefits and the drawbacks related to its use, is the most cited paper in *JMIR Rehabilitation and Assistive Technologies* [3], reflecting the increasing prominence of telerehabilitation, especially since the COVID-19 pandemic.

Approximately 2.4 billion people have a health condition that would benefit from or need rehabilitation [1,4]. Notably, the need for rehabilitation is estimated to increase as people live longer and with more chronic conditions and disability. There are substantial unmet needs in some low- and middle-income countries, with more than 50% of individuals not receiving the rehabilitation services they require. Conflicts, natural disasters, and disease outbreaks can increase these rehabilitation needs and disrupt existing services. Global needs remain unmet due to various factors, including a lack of available rehabilitation services outside urban areas, long waiting times, ineffective and underutilized referral pathways to rehabilitation, and lack of resources, including equipment and assistive technologies [1].

## Scope

*JMIR Rehabilitation and Assistive Technologies* focuses on pragmatic yet rigorous and impactful science that reports on the development, implementation, and evaluation of health innovations and interventions as well as emerging technologies in the field of rehabilitation. These innovations may also relate to a program such as a self-management intervention, clinical pathway, or device. Furthermore, we are interested in submissions that describe the need for rehabilitation interventions and innovations (eg, gaps in the transition from acute care to rehabilitation). We also welcome original research articles, review articles, viewpoints, or research letters [5] related to methodological advances in the study of rehabilitation and its assistive technologies. In particular, we are interested in papers that engage relevant knowledge users (eg, patients, families, etc) in developing, implementing, and evaluating these health innovations and interventions and emerging technologies. Mixed methods studies are highly relevant for studying the complexities of rehabilitation [6] and thus are also welcomed submissions. Consistent with the field of rehabilitation, we believe that *JMIR Rehabilitation and Assistive Technologies* is a venue for publishing interdisciplinary research between, for example, rehabilitation clinicians, scientists, and relevant knowledge users, including patients and families. Similarly, JMIR Publications, one of the first open access publishers, aims to reach wide audiences.

This engagement of multidisciplinary experts and community members will advance scientific knowledge and innovative care for rehabilitation services, and we look forward to your submissions to *JMIR Rehabilitation and Assistive Technologies.*
